# The Relationship between Family Violence and Self-Control in Adolescence: A Multi-Level Meta-Analysis

**DOI:** 10.3390/ijerph15112468

**Published:** 2018-11-05

**Authors:** Yayouk E. Willems, Jian-Bin Li, Anne M. Hendriks, Meike Bartels, Catrin Finkenauer

**Affiliations:** 1 Department of Biological Psychology, Vrije Universiteit Amsterdam, van der Boechorststraat 1, 1081 BT Amsterdam, The Netherlands; a.m.hendriks@vu.nl (A.M.H.); m.bartels@vu.nl (M.B.); c.finkenauer@uu.nl (C.F.); 2 Amsterdam Public Health Research Institute, Vrije Universiteit Amsterdam, van der Boechorststraat 7, 1081 BT Amsterdam, The Netherlands; 3 Department of Interdisciplinary Social Science, Universiteit Utrecht, Heidelberglaan 1, 3508 TC Utrecht, The Netherlands; 4 Department of Early Childhood Education, The Education University of Hong Kong, 10, Lo Ping Road, Tai Po, New Territories, Hong Kong, China; 5 Center for Child and Family Science, The Education University of Hong Kong, 10, Lo Ping Road, Tai Po, New Territories, Hong Kong, China; 6 Neuroscience Amsterdam, De Boelelaan 1085, 1081 HV Amsterdam, The Netherlands

**Keywords:** family violence, self-control, meta-analysis, adolescence

## Abstract

Theoretical studies propose an association between family violence and low self-control in adolescence; however, empirical findings of this association are inconclusive. The aim of the present research was to systematically summarize available findings on the relation between family violence and self-control across adolescence. We included 28 studies with 143 effect sizes, representing more than 25,000 participants of eight countries from early to late adolescence. Applying a three-level meta-analysis, taking dependency between effect sizes into account while retaining statistical power, we examined the magnitude and direction of the overall effect size. Additionally, we investigated whether theoretical moderators (e.g., age, gender, country), and methodological moderators (e.g., time lag between family violence and self-control, informant) influenced the magnitude of the association between family violence and self-control. Our results revealed that family violence and self-control have a small to moderate significant negative association (*r* = −0.191). This association did not vary across gender, country, and informants. The strength of the association, however, decreased with age and in longitudinal studies. This finding provides evidence that researchers and clinicians may expect low self-control in the wake of family violence, especially in early adolescence. Recommendations for future research in the area are discussed.

## 1. Introduction

Family violence—relational escalations in which one or more family members engage in verbal or physical violence—is common and has tremendous costs for individuals, communities and society. Individuals exposed to family violence show increased vulnerability to decrements in physical, mental, and social wellbeing across the lifespan [1,2,3]. It is a particularly harmful risk factor during adolescence, as family violence may jeopardize not only adolescents’ current wellbeing, but also their wellbeing as adults, and even the wellbeing of their future children [4,5]. Importantly, experiencing family violence predicts adolescents’ use of violence themselves, generating a vicious circle of violence from one generation to the next [6,7]. Although there is a consistent link between family violence and adverse outcomes for adolescents, development of effective prevention and intervention strategies would benefit from more knowledge on the specific processes underlying this link. 

Recent theoretical studies propose that self-control plays a key role in the family violence—adverse outcome link because of its foundational function in regulating behavior, emotions, and cognition [8,9]. Family violence may decrease adolescents’ self-control, and this decrease, in turn, is likely to carry over to cause adverse outcomes in other domains such as school, with peers, and in romantic relationships. Moreover, lowered self-control as a result of repeated exposure to family violence could make adolescents more likely lose self-control in stressful situations [10], thereby exacerbating violence within their family. Empirical evidence of these two theoretical core propositions, however, has produced mixed results. To illustrate, some studies find a significant association [11], while others show support for a cross-sectional and a longitudinal link between family violence and low adolescent self-control [12], and again other studies find a cross-sectional but not a longitudinal association [13,14], or find an effect from low self-control to family violence but no evidence for the reverse relation [15,16]. To shed light on the relation between family violence and self-control, this paper aims to summarize and quantify the association between family violence and self-control across adolescence through applying a three-level meta-analysis.

### 1.1. Conceptualization of Self-Control

Self-control involves the ability to initiate desirable actions and behaviors (e.g., finish homework, concentrate in class, achieve goals), and the capacity to inhibit undesirable impulses (e.g., suppress procrastination, overcome temper tantrums, avoid rule breaking; [17,18]). Self-control is an important concept within diverse research traditions, with criminologists and social psychologists embracing the term self-control, developmental psychologists using the terms effortful control, and clinical psychologists preferring the term self-regulation [18]. Empirical research shows that these terms collectively tap into the capacity to alter unwanted impulses and behavior and bring them into agreement with standards [19,20,21,22,23].

The capacity to perform self-control is of specific importance to adolescents. The teenage years are marked by a range of normative biological and social challenges [24], including increases in risk-taking behavior [25], and social reward seeking [26]. Low self-control hinders adolescents’ capacity to deal with these challenges. For example, adolescents with low self-control are less happy, have more negative social interactions, perform worse in school, and are more likely to get involved in oppositional behaviors and substance use than adolescents with high self-control [27,28,29,30]. Together, these findings highlight the importance of self-control during adolescence for healthy development across the lifespan.

### 1.2. The Relationship between Family Violence and Self-Control

Family violence is defined as destructive conflict within the family that is violent, frequent, and harmful. We conceptualize family violence as conflict that is frequent, involves verbal and/or physical overt aggression, and conflict that is rancorous or hostile in form and content and comprises multiple family members [31,32]. There are different pathways by which family violence may affect self-control. Family violence induces emotional stress in adolescents, resulting in behavioral, physiological, and cognitive dysregulation and lower self-control [33,34,35]. Additionally, studies show that family violence is a strong predictor of sleep problems, which, in turn, predicts self-control problems [35,36,37]. Rumination as a result of violent interaction is also likely to reduce self-control [38].

Moreover, studies suggest that family violence decreases self-control indirectly through processes associated with the family or the household. For example, family violence is predictive of more harsh discipline and less parental warmth and acceptance, limiting adolescents’ opportunities to learn through social observation how to manage their impulses and emotions [32,39]. Similarly, in families with family violence studies report lower parent-child relationship quality and lower sibling relationship quality which, in turn, undermines adolescents’ ability to develop self-controlled behavior [40,41,42]. These findings are consistent with the suggestion that family violence is negatively related to adolescents’ self-control at the within person level (stress, sleep, rumination) and through processes associated with the family and living conditions (parenting, family relationships).

Adolescents, nonetheless, are not passive recipients of their environment and some recent research suggests that adolescents with low self-control may evoke or maintain violence within the family. Adolescents with low self-control are more likely to undermine parental rules, which spurs parents to show over-controlling or hostile parenting strategies, exacerbating violence within the family [43]. This is in line with the behavior genetic literature, indicating that genetically influenced traits such as low self-control evoke harsh parenting responses, emphasizing the importance of taking child-driven effects into account [44,45]. Additionally, adolescents with low self-control are considered as less trustworthy by their family members and are less successful in de-escalating conflict [46,47]. Also, individuals with low self-control are more likely to show aggressive behavior in close relationships [48,49]. As such, the association between family violence and self-control can be understood as a transactional or reciprocal process, where contextual factors (family violence) affect the development of adolescents (self-control), and adolescents’ behavior evokes or maintains the context in which they develop.

In sum, in order to better understand the association between family violence and self-control, it is important to investigate the magnitude and the directional effect from family context to adolescent and from adolescent to family context [8,50,51]. A meta-analysis including longitudinal studies allows researchers to pit these effects against each other. Longitudinal studies include (a) an effect size where family violence is measured at one time point and self-control is measured at a succeeding time point and/or, (b) an effect size where self-control is measured at one time point and family violence at a succeeding time point, (c) or both. A meta-analysis allows to examine the average magnitude of these different effect sizes respectively. 

### 1.3. Moderators of the Link between Family Violence and Self-Control

An additional key strength of a meta-analysis is that it allows researchers to examine potential boundary conditions under which the relation between family violence and self-control may vary in magnitude. The association may vary as a function of theoretical moderators, such as age, gender, or country, and as a function of methodological moderators, such as whether the correlation pertains to cross-sectional assessments or longitudinal assessments, or to the type of informant.

### 1.4. Theoretical Moderators

#### 1.4.1. Age

Research shows that youth of all ages are adversely affected by family violence, yet the magnitude of the effect may vary across adolescence [32]. Throughout adolescence, teenagers increasingly claim more autonomy. As a result, some researchers argue that the association between family violence and low self-control is stronger during early adolescence, when teenagers are on the verge of gaining independence but still rely on parental support, than in later adolescence, when other social contexts and socializing agents become increasingly important (e.g., peers, school, neighborhood, [52]). Other evidence, however, suggests that the association increases over the course of adolescence because older children are likely to have been exposed to violence for a longer period of time [31,53]. Accordingly, in this meta-analysis we will explore whether the association between family violence and self-control changes as adolescents grow older. 

#### 1.4.2. Adolescent Gender

Evidence suggests that the effects of family violence are equally harmful for boys and girls [32]. Differences between boys and girls do become apparent in the way they perceive family violence; boys are more likely to perceive violence as a personal threat, while girls are more likely to perceive it as a threat to the harmony of the family system [54]. As a result, some research suggests gender differences in the developmental trajectories of the association between family violence and self-control. Specifically, research found that for girls the association was stronger during adolescence while for boys it was stronger in early childhood [54]. This study will explore whether the association between family violence and self-control is moderated by adolescent gender.

#### 1.4.3. Country

In their discussion sections, studies on the family violence—self-control link often suggest that findings should be replicated in different populations and international contexts. While we do not have specific hypothesis regarding country effects, a meta-analysis allows us to explore whether the association between family violence and self-control varies across countries or cultures. Moving beyond the classical “West” versus “East” paradigm, existing meta-analyses apply the continuous and nuanced culture scores developed by Hofstede to examine differences between countries [55,56,57]. These scores allow researchers to rate countries according to their level of individualism, attitude towards unequal distribution of power, and focus on competition and achievement within society. Applying such scores as moderators allows us to explore whether the association between family violence and self-control is generalizable across countries, or whether it shows different patterns across cultural dimensions [58,59,60,61].

### 1.5. Methodological Moderators

#### 1.5.1. Time Lag between Family Violence and Self-Control

Studies investigating the association between family violence and self-control have applied concurrent and/or prospective study designs: some assessed a cross-sectional association between family violence and self-control whereas others examined a longitudinal association. The differences in the magnitude of cross-sectional versus longitudinal studies are, however, not well quantified. An earlier meta-analyses on the link between attachment and self-control across the lifespan found larger effect sizes for cross-sectional studies as compared to longitudinal studies [62]. In the same vein, this meta-analysis will explore whether the magnitude of the association between family violence and self-control differs with the time lag between family violence and self-control.

#### 1.5.2. Informants

The magnitude of the association between family violence and self-control could vary depending on methodological specifications, such as the way violence and self-control are assessed (e.g., parent report or adolescent self-report), and whether they are assessed by the same informant (e.g., both self-report or both parent report, [19]). For self-control, correlations between self-reports are on average stronger than correlations between self-reports and other reports [63]. As such, we explore whether the association between family violence and self-control differs depending on informant, and whether it differs when both are assessed by the same person. 

### 1.6. The Present Study

While there is evidence for the link between family violence and self-control, empirical evidence regarding the magnitude and the direction of the effect remains inconclusive. The aim of the present study is to ‘take stock’ of the published literature so far by applying a three-level meta-analysis. A meta-analysis is ideal to summarize the published literature, because it allows for aggregating diverse individual study results to identify the overall mean effect and investigate the role of possible moderators on the magnitude of this effect. Doing so allows us to (1) quantify the relationship between family violence and self-control across adolescence, (2) examine the influence of theoretical and methodological moderators, and (3) elucidate gaps and questions that require attention in future research.

## 2. Methods

### 2.1. Literature Search

We collected data through systematic database search of *ERIC*, *PsycInfo*, *Pubmed*, and *Web of Science* until September 2018, following the Preferred Reporting Items for Meta-Analyses (PRISMA) checklist. Search terms included family variables (parent* or mother* or father* or parental or maternal* or attachment* or family* or bond*), self-control variables (self-control or self-regulation or self-discipline or effortful-control), and adolescent variables (adolescent* or adolescence or teen* or youth* or child* or student* or undergraduate or emerging adult* or young adult*). We chose the adolescent age span from age 10 to 22 years, in order to capture the broad developmental range of teenage development [24].

In order to ensure extensive search outcomes, we applied search terms capturing broad family variables. First, when reporting on family violence, it is common to mention a family related keyword in the title or abstract (e.g., parent, adolescent, family). In our search, we included all studies that mentioned family related key words in the title or abstract for full text screening (e.g., parent, mother, father, parental, family, bond, adolescent, child). Second, in some studies family violence is not the key focus but included for exploratory analyses. By applying these broad terms, we were able to include studies that specifically focus on the family-self-control association and capture studies that have a different research question but include violence as an explorative variable or covariate. Third, some studies do not explicitly mention family violence in their abstract but apply measures assessing family violence (for example as a dimension of harsh parenting). Our extensive search allowed us to include a large number of studies and inspecting parenting measures thoroughly to detect studies including effect sizes on family violence and self-control.

**Figure 1:**
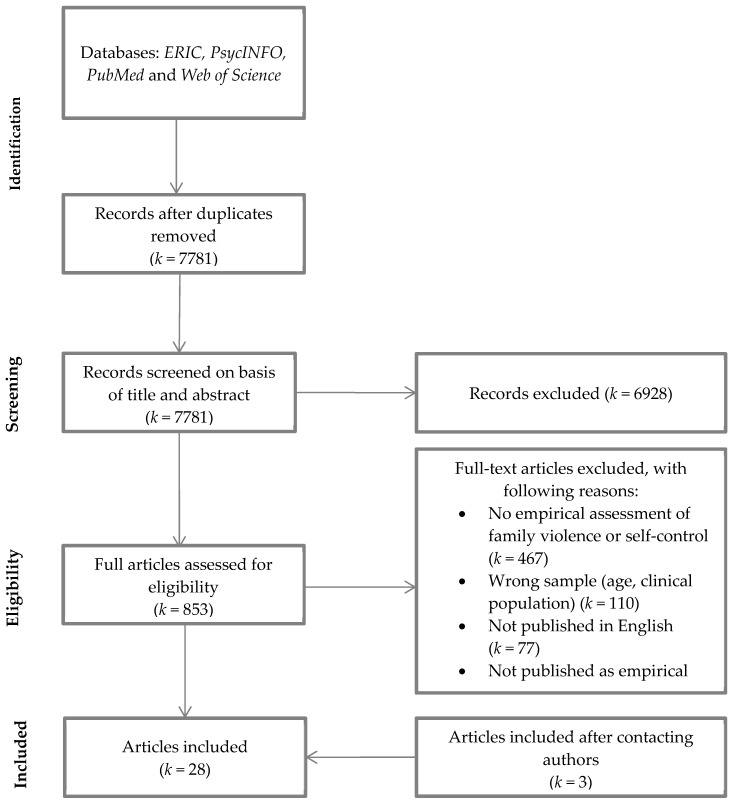
PRISMA flowchart used to identify studies for detailed analysis of family violence and self-control.

Studies were included if (1) the study included a correlation between family violence (on any relational level) and self-control, (2) the study included non-clinical samples, (3) the study was published in English, in a peer-reviewed journal, and (4) the age of the participating adolescents was between 10 and 22 years. We adopted this wide age range to explore the association from the start of puberty into solidification of adulthood [24,64] (see Figure 1 for the flowchart).

### 2.2. Selection of Studies

Our search yielded 7781 hits which, after removing duplicates of the multiple search engines and applying inclusion criteria to the title and abstract, resulted in 853 potentially relevant articles for full text screening. Of the 853 articles, *k* = 467 articles were excluded because they did not measure family violence or self-control (e.g., only mentioned the concept in their introduction or discussion without empirical assessment), *k* = 110 were excluded because they focused either on the wrong age or on a clinical population, *k* = 77 were excluded because the full text was not published in English, and *k* = 163 were excluded because they were not published as an empirical article (e.g., dissertations, student theses, or conference abstracts). Additionally, *k* = 11 articles did assess the association between family violence and self-control but did not include a correlation table. Authors of these articles were contacted, resulting in an additional *k* = 3 articles to include in the present meta-analysis. In total, 28 studies met the abovementioned inclusion criteria and were included in the present meta-analysis (see Figure 1 for the flowchart).

We collected relevant information of the studies and organized them according to a detailed coding scheme [65]. This coding scheme included study descriptors (e.g., author names, title, year of publication, data collection details, sample size), moderator variables (e.g., time lag between family violence and self-control, age, country, informant), and the correlation between family violence and self-control (retrieved from correlation tables or provided by contacted authors).

### 2.3. Inter-Rater Agreement

To calculate inter-rater reliability, the first two authors double coded 17% of the articles (*k* = 148, of the *k* = 853 full articles assessed for eligibility). This resulted in a good inter-rater reliability, reflected in high intra-class correlations for continuous variables (ranging between 0.78 for age to 0.99 for sample size) and high Cohen’s Kappa for categorical variables (ranging between 0.86 for informant, and 1.00 for country of the study). In case of disagreement, in-depth discussions were held to reach agreement on the specific content of the article. Full consensus was reached on all variables, providing us the confidence to divide the remaining articles between the two of us. The remaining 83% was divided equally among both authors.

### 2.4. Theoretical Moderators

#### 2.4.1. Age

We coded age at assessment continuously. For studies not reporting age but school grade, the average age of students in that school year was coded. For example, when the study mentioned adolescents were in sixth grade in the USA, we coded mean age as 11.5 years.

#### 2.4.2. Adolescent Gender

The proportion of boys and girls participating in the study was continuously coded, coding the percentage of girls in the sample (which could range from 0% to 100%). For example, studies with 60 girls and 40 boys was scored as 60%. Studies reporting effect sizes separately for boys and girls, adolescent gender was coded as 0% and 100%, respectively. 

#### 2.4.3. Country

The influence of country was assessed by applying the Hofstede dimensions, which are frequently applied in meta-analyses to examine the generalizability of an association across cultures [55,56,57]. Countries were coded according to Hofstede’s individualism score, power distance score, and femininity-masculinity score ([55], or see https://geert-hofstede.com/countries.html): (i) the individualism score reflects the extent to which identity is based on self-orientation and emphasis on individual achievement and initiative rather than collectivism (i.e., identity based on group orientation with emphasis on social system and belonging); (ii) power distance reflects a country’s attitude towards the unequal distribution of power; (iii) masculinity reflects the extent to which a society is driven by competition, and achievement rather than by an emphasis on quality of life.

### 2.5. Methodological Moderators

#### 2.5.1. Time Lag between Family Violence and Self-Control

For every study, we coded the time lag between the assessment of family violence and the assessment of self-control continuously in years (starting with a code of 0 for cross-sectional studies).

#### 2.5.2. Informants

For every effect size, we coded whether family violence was assessed by adolescents themselves (1 = *self-report*), by someone else such as one of the parents (2 = *other-report*), or whether the measure was a composite of different informants (3 = *composite*). Similarly, informant of the self-control measure was coded according to the reporting informant (1 = *self-report*, 2 = *other-report, such as parent report*, 3 = *composite of measures*, for example combination between self- and parent-report). 

Furthermore, studies were coded with 1 = *consistent*, when family violence and self-control were assessed by the same informant (e.g., both by adolescents themselves) and coded with 2 = *inconsistent*, when family violence and self-control were assessed by different informants (e.g., family violence by parents and self-control by adolescents themselves). Important to note is that when both consisted of composite measures, specific attention was paid to check whether these composite scores comprised of the same informants. For example, a code of 1 was given when violence was measured with a composite score consisting of self-report and mother report and self-control was also measured with a composite score consisting of self-report and mother report. However, when violence was measured with a composite score of self-report and parent report and self-control with a composite score of self-report and teacher-report, a score of 2 was given.

#### 2.5.3. Effect Sizes

We obtained Pearson correlation coefficients to examine the strength of the association between family violence and adolescent self-control. The correlations were either derived from the studies or retrieved upon request if they were not present in the published paper. For consistency, we recoded effect sizes in which self-control was assessed as ‘lack of self-control’ or ‘low self-control’. For normalization and standardization, correlations were transformed into Fisher’s Z scores ES_Z_ [65]. The ES_Z_ were the input for the analyses; after the analyses they were transformed back to *r* for interpretation (see Appendix A). Categorical moderator variables were dummy-coded with *k*—1 dummy variables [66].

#### 2.5.4. Publication Bias

To take the possibility of publication bias into account, we created a funnel plot and performed an Egger’s test on the effect sizes. The funnel plot allowed us to inspect the distribution of the effect sizes by displaying each individual effect size in a figure with the effect sizes on the horizontal axis and study precision as a function of standard errors on the vertical axis [67]. Publication bias would occur if the funnel plot displayed an asymmetrical distribution. In order to formally test whether there was an asymmetrical distribution of effect sizes, we conducted an Egger’s regression test [67].

### 2.6. Data Analyses

We performed all our analyses in R version 3.4.2 [68], using the Metafor package [69]. Because most studies reported multiple effect sizes, there was a likely dependency between effect sizes derived from the same studies (e.g., these effect sizes are not independent as they are part of the same sampling process, study group, and study population). To take this dependency into account, we applied a three-level meta-analysis, an approach that allows us to use all available information (i.e., multiple effect sizes) [66,70,71].

The three-level model specifies the following levels of variance: (1) sampling variance of the effect sizes, (2) variance between effect sizes within studies using the same dataset, and (3) variance between studies [71]. Studies using the same dataset are treated as if they all come from the same study. In this approach, studies with multiple effect sizes will not necessarily be assigned more weight because the dependency between effect sizes is taken into account. In contrast to the “classic” meta-analytic approach, selecting only one effect size from a single study or averaging effect sizes within studies, the three-level meta-analysis allows to include all effect sizes while taking the dependency into account. Doing so enables researchers to retain a maximum of information and achieve greater statistical power [70,71,72]. To take into account possible dependency between studies using the same dataset, we used the number of independent studies (i.e., data sets) as the mode of analysis [66]. 

To examine the association between family violence and adolescent self-control and moderator effects, we performed the following analyses. First, we estimated the overall mean effect size of the association. Second, we assessed between-study and within-study variance using a likelihood ratio test, and partitioned the total variance into percentages for the sampling variance, variance within studies, and variance between studies, applying earlier proposed methods [70,71,72]. Third, based on whether there was evidence for heterogeneity among effect sizes, we performed univariate-moderator analyses. Fourth, we conducted multivariate moderator analyses to assessing significance of each moderator while considering other significant moderators to avoid multicollinearity problems in the analyses. The analyses were performed in line with earlier described procedures [66], estimating parameters using restricted maximum likelihood.

## 3. Results

### 3.1. Descriptives

The present meta-analysis included 28 studies reporting on the association between family violence and self-control. All included information is presented in an Excel table, facilitating opportunities for other scholars to use, update or extend our data for future research purposes (see Table S1, Supplementary Materials, contact the first authors to receive the R script). Family violence included measures of severe punishment, slapping/hitting, physical coercion, severe verbal fights within the family, heatedly shouting and criticizing within the family, expressive anger and frequency of violence. It included general family violence, marital violence and parent-child violence. Self-control included measures of self-control, self-regulation, self-restraint, effortful control and persistence. 

Of the 28 studies, 25 reported on independent studies, including 143 effect sizes and a total sample size of *N* = 26,214. Studies were published in a wide range of journals, for example in the Journal of Family Studies, Journal of Youth and Adolescence and Journal of Crime and Delinquency, and were published between 1990 and 2017. Most studies were conducted in the USA, followed by studies conducted in Asia and Europe. Age of the participating adolescents ranged between 10.00 and 21.70 years, with a mean age of 13.41 years (See Table 1 for more details). Most studies reported cross-sectional associations (26 studies, 104 effect sizes), with 5 studies (39 effect sizes) reporting longitudinal associations from family violence to self-control. 

Studies focusing on the effect from self-control to family violence were scarce. Of the 28 included studies, we only identified three studies reporting longitudinal associations where self-control was measured first and family violence at a subsequent time point. While some argue three studies are enough to meta-analyze an effect, parameter estimates are poor when the number of studies is below five [73]. As a result, we could not meta-analyze the magnitude of the effect from self-control to family violence nor could we address the question regarding reverse causality, namely whether the magnitude of the directional effects differed. The results, therefore, only present cross-sectional effect sizes and longitudinal effect size from family violence to self-control.

### 3.2. Publication Bias

**Figure 2:**
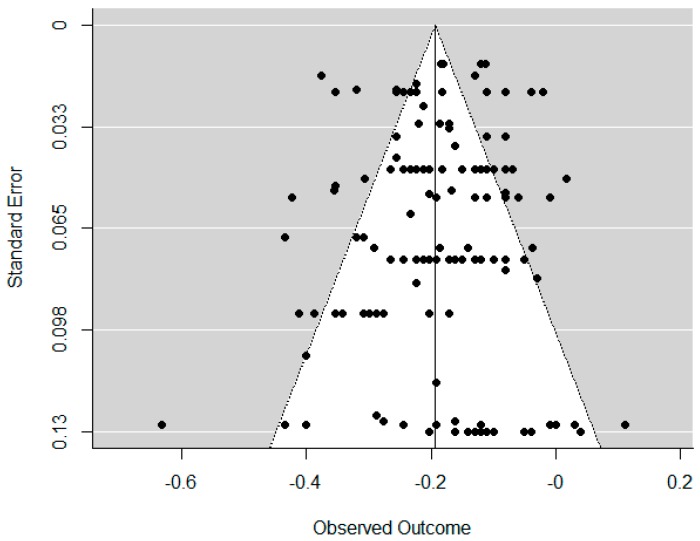
Funnel plot.

As shown in Figure 2, the distribution of the effect sizes in the funnel plot appeared to be symmetrical. In addition, the Egger’s test was non-significant Z = −0.994, *p* = 0.320. This suggested that there was no publication bias in the present meta-analysis. 

### 3.3. Overall Effect Size

We found a negative small to medium significant overall effect size for the association between family violence and adolescent self-control (ES_Z_ = −0.194, S.E. = 0.015, t = −12.982, *p* < 0.001, 95% CI = (−0.223, −0.164), *r* = −0.191). This indicated that more family violence is significantly associated with lower adolescent self-control. 

### 3.4. Variance of the Overall Effect Size

There was significant variance within studies (estimate = 0.006, *p* < 0.001) and between studies (estimate = 0.003, *p* < 0.001). Partitioning the variance into percentages for the three levels revealed that the variance at the sampling level was 13.62%, variance within studies using the same dataset was 61.20%, and variance between studies using different datasets was 25.18%. These results, in addition to the significant residual heterogeneity of the overall effect size (QE(142) = 1017.972, *p* < 0.001), indicated appropriateness for further moderator analyses.

### 3.5. Moderator Analyses

We performed univariate moderator analyses; Table 2 displays the statistics for the results. Significant moderators were age (QE(140) = 901.684, *p* < 0.001; Omnibus test: F(1, 140) = 8.913, *p* = 0.003) and time lag between family violence and self-control (QE(140) = 836.663, *p* < 0.001; Omnibus test: F(1, 140) = 8.367, *p* = 0.004). We explored the possibility that age as a moderator would show a non-linear pattern. Comparing age with a linear pattern versus age with a non-linear pattern indicated the linear pattern to fit the data best (cf. lower Akaike Information Criterion (AIC) value for the linear pattern). The other moderators were not significant, including adolescent gender, Hofstede’s scores, informant family violence, informant self-control, and consistency in informants. 

Based on the significant moderators found in the previous analyses (age and time lag between family violence and self-control), we conducted a follow-up comparison as summarized in Table 3. Regarding age (centered at age 10), we found a significant effect (β_0_ = −0.249, S.E. = 0.024, t = −10.288, *p* < 0.001, 95% CI = (−0.297, −0.202), *r* = −0.243), and a significant and positive slope (β_1_ = 0.015, S.E. = 0.005, t = 2.985, *p* < 0.001, 95% CI = (0.005, 0.025)). This indicates a decrease in the magnitude of the association as adolescents get older (the constant is negative, and the positive slope will thus mitigate the starting value).

Regarding time lag between family violence and self-control, we found a significant effect (β_0_ = −0.201, S.E. = 0.015, t = −13.505, *p* < 0.001, 95% CI = (−0.230, −0.171), *r* = −0.198) and a significant positive slope (β_1_ = 0.036, S.E. = 0.012, t = 2.893, *p* = 0.004, 95% CI = (0.011, 0.061)). This indicates that the longer the time in-between measurements, the smaller the effect size.

### 3.6. Multiple Moderator Model

We conducted a multiple moderator model including both significant moderators from the univariate moderator analyses to assess their unique contribution (i.e., time lag between family violence and self-control, and age). The results of this multivariate model are summarized in Table 4. The significant omnibus test (F(2, 139) = 8.459, *p* < 0.001) suggested that at least one of the parameter estimates of the moderators significantly deviated from zero. Subsequent ANOVA tests indicated that both age and time lag had unique moderating effects on the relationship between family violence and self-control.

## 4. Discussion

In the present meta-analysis, we synthesized research on the association between family violence and self-control across adolescence. We included 28 studies, conducted in eight countries, containing 143 effect sizes, with a total sample size of *N* = 26,214. The findings from the three-level meta-analysis revealed that family violence and self-control are significantly, small to moderately, negatively associated (*r* = −0.191). This indicates that family violence and low self-control coincide. 

### 4.1. Moderators

Moderator analyses revealed that the association between family violence and low self-control did not differ significantly across country, adolescent gender, and informant. We did find a linear moderator effect for age; the magnitude of the association between family violence and self-control decreased over the course of adolescence. This finding suggests that adolescents gradually transform from parent-dependent to self-sustaining independent individuals [52,77,78]. As a result, the influence of family factors such as family violence on adolescents may decrease, while the role of other contextual factors may increase. In the context of adolescence, this could indicate that the peer context becomes of increasing importance, perhaps buffering the negative effects experienced within the family [79]. 

We also found a moderator effect for the time between the measurement of family violence and self-control, with decreasing effect sizes for studies with a longer time lag between the assessment of family violence and subsequent self-control. This is in line with earlier methodological studies on the link between family factors and self-control, similarly indicating that the association is stronger when measured concurrently as compared to longitudinal assessments [62]. This is likely a result of more intervening processes taking place along the way, waning the direct effects of family violence on adolescent self-control.

It is important to note that we should be cautious in interpreting the direction of the effect. The association between family violence and self-control is likely to reflect a transactional process by which family violence and adolescent self-control mutually affect each other [44,52,78,80]. As such, family violence is likely to decrease self-control, which is in turn likely to evoke or exacerbate family violence [8,9]. The present meta-analysis revealed that most of the longitudinal studies included an effect from family to adolescent, without examining the reverse effect. While the results of the present meta-analysis provide an interesting starting point suggesting a link between family violence and self-control, future research on the links between family violence, self-control and psychosocial problems in a time sequential design are recommended (for example through random intercept cross-lagged panel models [81]).

### 4.2. Implications

Adolescents exposed to family violence show heightened vulnerability to decrements in physical, mental, and social wellbeing. Although linkages between family violence and various problems are well-established, the specific processes underlying these associations are poorly understood. Recent theoretical work proposes self-control to play an important role in explaining these links [8,9]. On the one hand low self-control may function as a possible mechanism because it is affected by family violence and contributes to maintaining violence. On the other hand, low self-control is reliably related to poorer physical, mental, and social health and wellbeing [30,82]. Supporting these theoretical suggestions [8,9], we found a significant association between family violence and self-control across adolescence, suggesting that self-control may play an important role in the link between family violence and adverse outcomes. As such, researchers and clinicians can expect low self-control in the presence of family violence, as opposed to treating low self-control and family violence as separate problems. For instance, family-based therapies targeting both family violence and self-control may well result in increased adolescent well-being and better family functioning, yet controlled trials are necessary to confirm this suggestion.

## 5. Limitations

First, we did not distinguish between interparental, parent-child, sibling-child, and parent-sibling violence, because most studies reported on family violence as a general construct without specifically specifying the family (sub)relationships involved in the conflict. Few studies provided in-depth details to distinguish between different relational levels at which the violence occurred. While both witnessing violence and experiencing violence are considered as detrimental for adolescents [83], further research is recommended to more specifically describe, measure, and compare different types of violence and their association with self-control in adolescence [32].

Second, it is important to acknowledge that, when investigating interactions within families, not only environmental but also genetic factors play a role [44]. This is evidenced by studies reporting on the intergenerational transmission and the heritability of family violence [84,85], and the intergenerational transmission and the heritability of self-control [33,63]. As a result, it may be that the observed association is partly explained by common genetic factors that simultaneously influence both family violence and self-control [44,86]. To paint a more complete picture of the association, future studies that integrate genetically sensitive designs investigating both environmental and genetic influences on the association between family violence, self-control and psychosocial problems and wellbeing would be particularly helpful. 

## 6. Conclusions

Self-control—the capacity to regulate thoughts, emotions, and behavior—is a core component of healthy adolescent development. Results from the current meta- analysis indicate that family violence and adolescent self-control are negatively related, especially among younger adolescents. Because low self-control and family violence are reliably related to poorer health and wellbeing across the lifespan, these findings underscore the importance of considering both contextual and individual factors in treatment and policy addressing family violence. Although family violence is linked with adolescent self-control, and this link is not affected by a broad variety of moderators, we did find that the effects are stronger in studies with a shorter time delay. The meta-analysis also identified important gaps in our knowledge on the influence of genetic factors and reverse causality thereby providing promising inroads to enhance our understanding of the association between family violence and adolescent self-control.

## Figures and Tables

**Table 1:** Description.

Variable	Characteristics	Descriptives
Studies included	*K* studies	28
	*K* independent studies	25
	*N* effect sizes	143
Publication year	Range	1990–2017
Journals	Range	20 different journals, e.g., Journal of Crime and Delinquency, Journal of Family Psychology, Journal of Youth and Adolescence
Dataset	Including	Flourishing families project, Healthy Families America (HFA) San Diego study, Longitudinal Study of Australian Children (LSAC), NICHD Study of Early Child Care and Youth Development (SECCYD), the Family and Community Health Study (FACHS)
Sample Size	Total sample size	26,214
	Min sample size	65 [74], 120 [16]
	Max sample size	3797 [75], 6429 [76]
Age	Mean	13.41
	Min–Max	10–21.7
Adolescent gender	Overall balanced	87 effect sizes (*k* = 20)
	>60% boys	22 effect sizes (*k* = 7)
	>60% girls	34 effect sizes (*k* = 6)
Countries	Australia	1
	Hong Kong	2
	Germany	1
	Israel	1
	South Korea	1
	Switzerland	1
	UK	1
	USA	20
Hofstede individualism	Range	18 (South-Korea)–91 (USA)
Hofstede power distance	Range	13 (Israel)–68 (Hong Kong)
Hofstede masculinity	Range	39 (South-Korea)–70 (Switzerland)
Time lag between family violence and self-control	Cross-sectional Longitudinal effect	104 effect sizes (*k* = 26)39 effect sizes (*k* = 5)
	Average longitudinal delay	1.30 years
Informant family violence	Self-report	79 effect sizes (*k* = 18)
	Other report	6 effect sizes (*k* = 4)
	Composite	54 effect sizes (*k* = 6)
Informant self-control	Self-report	56 effect sizes (*k* = 18)
	Other report	59 effect sizes (*k* = 7)
	Composite	20 effect sizes (*k* = 3)
Consistency	Consistent	67 effect sizes (*k* = 17)
	Inconsistent	76 effect sizes (*k* = 13)

**Table 2:** Assessing moderators: The QE statistics illustrating residual heterogeneity, and the Omnibus to test the effect of the moderators on the family violence-self-control association.

Moderator	*k_i_*	*N* ES	Omnibus Test	*p*	Variance Level 2	Variance Level 3	QE (df)	*p*
Age	24	142	F(1, 140) = 8.913 **	0.003	0.005 **	0.003 **	901.684 (140)	<0.001
Adolescent gender	25	143	F(1, 141) = 0.319	0.573	0.006 **	0.002 **	1011.77 (141)	<0.001
Hofs. Individualism	25	143	F(1, 141) = 0.195	0.659	0.006 **	0.003 **	1017.332 (141)	<0.001
Hofs. Power distance	25	143	F(1, 141) = 0.997	0.320	0.078 **	0.005 **	1009.720 (141)	<0.001
Hofs. Masculinity	25	143	F(1, 141) = 0.049	0.825	0.006 **	0.003 **	999.909 (141)	<0.001
Time lag	24	142	F(1, 140) = 8.367 **	0.004	0.006 **	0.002 **	836.663 (140)	<0.001
Informant fv	23	139	F(2, 136) = 0.377	0.687	0.006 **	0.003 **	898.725 (136)	<0.001
Informant sc	25	135	F(2, 132) = 0.326	0.326	0.006 **	0.003 **	923.373 (132)	<0.001
Consistency	25	143	F(1, 141) = 0.214	0.644	0.006 **	0.002 **	1016.895 (141)	<0.001

Note: **—*p* < 0.01, *k_i_*—number of independent studies, *N* ES—number of effect sizes, Hofs—Hofstede’s scores. Time lag—time lag between family violence and self-control, fv—family violence, sc—self-control.

**Table 3:** Univariate analyses presenting slopes of the significant moderators.

Moderators	*N* ES	ES_Z_	SE	*T*	95% CI	*p*	*r*
Age	142	−0.249	0.024	−10.288	(−0.297, −0.202)	<0.001	−0.244
		0.015	0.005	2.985	(0.005, 0.025)	0.003	
Time lag	142	−0.201	0.015	−13.505	(−0.230, −0.171)	<0.001	−0.201
		0.036	0.012	2.893	(0.011, 0.061)	0.004	

Note: Age was centered at age 10 (the minimum age of the present meta-analysis). Time lag = time lag between family violence and self-control.

**Table 4:** Results for the multiple moderator model.

Moderator Variables	ES_Z_ (SE)	95% CI	*t*-Statistic	*p*-Value
Intercept	−0.248 (0.022) **	(−0.291, −0.204)	−11.334	<0.001
Age	0.013 (0.005) **	(0.004, 0.022)	2.793	0.006
Time lag	0.033 (0.012) **	(0.009, 0.057)	2.725	0.007
Omnibus test:	*F*(2, 139) = 8.459 **			
Variance level 2	0.005 **			
Variance level 3	0.002 *			
*N* ES	142			

Note: *—*p* < 0.05,**—*p* < 0.01. Time lag—time lag between family violence and self-control.

## Supplementary Materials

The following are available online at http://www.mdpi.com/1660-4601/15/11/2468/s1, Table S1: Reviewed Studies, Sample Characteristics, Methods of Assessment, and Effect Sizes. References [11,12,13,14,16,74,75,76,87,88,89,90,91,92,93,94,95,96,97,98,99,100,101,102,103,104,105,106] are included in the supplementary meta-analysis.

## Appendix A

The Fisher’s transformation of *r* was done using the following formula: ES_Z_ *=* $\frac{1}{2}\log_{e}\left[\frac{1+r}{1-r}\right]$. Any ES_Z_ can be transformed back into standard correlation form using the inverse of the ES_Z_ transformation using the following formula: $r=\frac{e^{2\mathrm{ESZ}}-1}{e^{2\mathrm{ESZ}}+1}$ [63].
